# Retraction: Biochemical properties and biological potential of *Syzygium heyneanum* with antiparkinson’s activity in paraquat induced rodent model

**DOI:** 10.1371/journal.pone.0360894

**Published:** 2026-10-20

**Authors:** The PLOS One Editors

Following the publication of this article [1] concerns were raised about results published in Figs 2 and3 and Tables 4-6. Specifically,

- The following panels appear similar, despite being used to represent different experimental conditions:
  - Fig 2 CG Liver of [1] and Fig 2 CG:Liver of [2].
  - Fig 2 CG Lungs of [1] and Fig 2 CG:Lung of [2].
  - Fig 2 TR Heart of [1] and Fig 2 TR:Heart of [2].
  - Fig 2 TR Lungs of [1] and Fig 2 TR:Lung of [2].
  - The Fig 3 CG panel of [1] and the CG panel in unpublished material submitted for [3, retracted in 4].
- In Tables 4-6, the reported values for the standard error of the mean appear unusually repetitive.

Regarding the concerns with similarities between results presented in Fig 3 of [1] and unpublished material submitted with [3, retracted in 4], the corresponding author stated that errors were made during the figure preparation of the images presented in the original version of [3, retracted in 4], which were corrected in subsequent revisions of the manuscript. Regarding the concerns with similarities between material in this article and the material published in [2], the corresponding author stated that the material in [2] was used without the data owner’s authorization.

The corresponding author provided the individual-level data underlying the graphs and tables presented in this article. Editorial assessment of these data raised additional concerns about the integrity and reliability of these data.

In light of the concerns observed with the individual-level data underlying this study, the *PLOS One* Editors retract this article.

MA did not agree with the retraction. ZC responded but did not clarify either agreement or disagreement. MS, HT, US, SAB, ASehar,and ASethi either did not respond directly or could not be reached.
